# The mosaic distribution pattern of two sister bush‐cricket species and the possible role of reproductive interference

**DOI:** 10.1002/ece3.6086

**Published:** 2020-02-08

**Authors:** Martina Dorková, Anton Krištín, Benjamín Jarčuška, Peter Kaňuch

**Affiliations:** ^1^ Institute of Forest Ecology Slovak Academy of Sciences Zvolen Slovakia; ^2^ Faculty of Ecology and Environmental Sciences Technical University in Zvolen Zvolen Slovakia

**Keywords:** coevolution, cross‐mating, home ranges, hybridization, Orthoptera, precopulatory behavior

## Abstract

Reproductive interference can shape regional distribution patterns in closely related species, if prezygotic isolation barriers are weak. The study of such interaction could be more challenging in nuptial gift‐giving species due to the direct nutritional effects on both sexes of both species during copulation. We mapped the distribution of two sister bush‐cricket species, *Pholidoptera aptera* and *Pholidoptera transsylvanica*, at the northern margin of their overlapping ranges in Europe, and with a behavioral experiment, we tested the possibility of heterospecific mating. We found a very rare coexistence of species locally (0.5%, *n* = 391 sites) with mostly mutually exclusive distribution patterns, resulting in a mosaic pattern of sympatry, whereas they occupied the same climate niche in forest‐dominated mountain landscape. Over 14 days of a mating experiment with seven mixed groups of conspecifics and heterospecifics (*n* = 56 individuals in total), the number of received spermatophores per female was 3–6 in *P. aptera* and 1–7 in *P. transsylvanica*. In total, we found 8.1% of heterospecific copulations (*n* = 99 transferred spermatophores with genetic identification of the donor species), while we also confirmed successful transfer of heterospecific sperms into a female's reproductive system. Because bush‐cricket females also obtain required nutrition from a heterospecific spermatophylax what should increase their fitness and fecundity, we suggest that their flexibility to mate with heterospecifics is beneficial and drives reproductive interference. This may substantially limit the reproductive success of the less frequent species (*P. transsylvanica*), coupled with eventual detrimental effects from hybridization, and result in the competitive exclusion of that species from their areas of coexistence.

## Graphical abstract

We found a very rare coexistence of *Pholidoptera aptera* and *Pholidoptera transsylvanica* with mostly mutually exclusive distribution patterns, whereas they occupied the same climate niche in forest‐dominated mountain landscape. Experimental trials revealed 8% of heterospecific copulations, while successful transfer of heterospecific sperms into a female's reproductive system was also confirmed. Because females also obtain required nutrition from a heterospecific spermatophylax, we suggest that this reproductive interference may result in the competitive exclusion of the inferior species from their areas of coexistence.

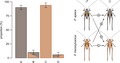

## 1 INTRODUCTION

The study of interactions between closely related taxa which occupy a sympatric range is important for understanding the evolutionary adaptations and isolation mechanisms that lead to their coexistence or divergence (Chesson, 2000; Hewitt, 2001). A specific spatial niche, habitat or behavior, often limits species interactions and prevents their local coexistence (Kádár, Fazekas, Sárospataki, & Lövei, 2015; Michalko, Košulič, Hula, & Surovcová, 2016; Pellissier et al., 2012; Schirmel & Fartmann, 2013). However, when species interact more closely, resource competition and especially reproductive interference (heterospecific mating) may hamper their coexistence even more, resulting in a mosaic pattern of sympatry (Gröning, Lücke, Finger, & Hochkirch, 2007; Hochkirch, Gröning, & Bücker, 2007). To learn how regional distribution patterns of such species are shaped, we should be interested in both the barriers that prevent coexistence and the factors that result in the competitive exclusion (demographic displacement) of the inferior species (Grether, Losin, Anderson, & Okamoto, 2009; Gröning & Hochkirch, 2008; Kyogoku, 2015; Wellenreuther, Larson, & Svensson, 2012).

Heterospecific mating, ranging from mating attempts to hybridization between sister species, has been demonstrated in a wide range of animal taxa (Gröning & Hochkirch, 2008), and it is also known in different genera of Orthoptera (e.g., *Allonemobius*, Gregory & Howard, 1993; *Pterophylla*, Barrientos‐Lozano, 1998; *Orchelimum*, Shapiro, 2000; *Tetrix*, Hochkirch, Deppermann, & Gröning, 2006, Gröning et al., 2007, *Chorthippus*, Vedenina, Kulygina, & Panyutin, 2007; *Poecilimon*, Lehmann, Siozios, Bourtzis, Reinhold, & Lehmann, 2011; *Aglaothorax*, Cole, 2016; *Phaneroptera*, own unpublished data). In Orthoptera, such occasional events suggest utilization of similar signal channels during mate recognition (Hochkirch et al., 2006) and/or the similar morphology of their sexual organs, which are used as stimulatory devices during copulatory courtship and for spermatophore transfer (Wulff, Kamp, Santos, Baumbach, & Lehmann, 2017; Wulff, Lehmann, Hipsley, & Lehmann, 2015). Copulation between related species shows no obvious behavioral differences to conspecific mating, as spermatophores, including sperms, are successfully transferred (Lehmann et al., 2011). If there is a weak genetic basis for postzygotic reproductive isolation, then potential interference and hybridization may have detrimental effects on the reproductive output of at least one of the species (Gregory & Howard, 1993; Gröning & Hochkirch, 2008; Shapiro, 2000). On the other hand, because the spermatophores of bush‐cricket males include a proteinaceous courtship meal (the spermatophylax) that is served to females during copulation, this interaction should also include direct nutritional effects on both sexes of both species (Vahed, 1998). Heterospecific nuptial gifts can be energetically beneficial for females, increasing their fitness and fecundity (Brown, 1997; Fedorka & Mousseau, 2002; Voigt, Kretzschmar, Speakman, & Lehmann, 2008), but they should be unprofitable for males, which expended resources to manufacture them (Costa‐Schmidt & Machado, 2012; Lehmann, 2012; Simmons, 1990; Vahed, 2007). Thus, reproductive interference between competing nuptial gift‐giving species may be even more entangled and interesting also from the view of the evolution of their distributional ranges (Tregenza, 2002).

**Figure 1:**
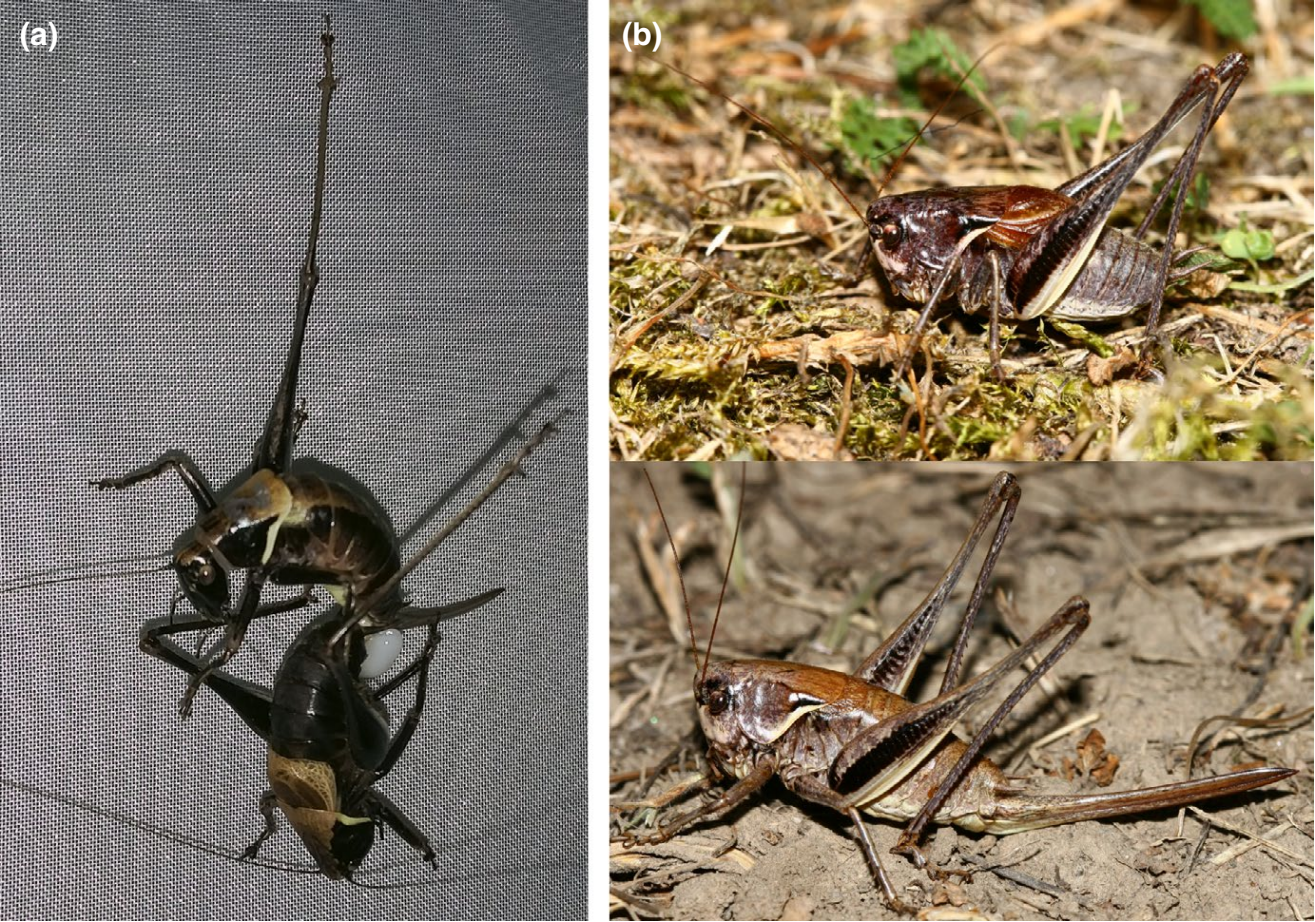
(a) A male (bottom) of *Pholidoptera aptera* attaches to the female's genital opening a spermatophylax (white mass) during mating experiment. (b) A male (top) and female (bottom) of *Pholidoptera transsylvanica* in Slovakia. Photographs by A. Krištín

The Alpine and the Transylvanian dark bush‐crickets, *Pholidoptera aptera* (Fabricius 1793) and *Pholidoptera transsylvanica* (Fischer, 1853), body length 17–25 and 19–30 mm, respectively (Figure 1), are related, morphologically similar, ground‐living and flightless Orthoptera species with European distribution (Chobanov et al., 2016; Harz, 1969; Hochkirch et al., 2016). The Transylvanian dark bush‐cricket is a species of European importance by the Habitats Directive of the EU (Krištín & Kaňuch, 2013), and both these large predatory bush‐crickets belong to species of preserved habitats where their behavior is still difficult to study due to cryptic life and low abundance (e.g., up to 5–15 males/ha; own data). The species extent of occurrence suggests a possible overlap of their ranges, mostly in the Carpathian Mountains (Chobanov et al., 2016; Hochkirch et al., 2016; Figure S1). However, there is almost no evidence on their coexistence at the same sites. In Slovakia, at the northern margin of their ranges, only two old records of coexistence were known until now (Chládek, 2003). Both species are considered to be dwellers at forest edges, in open forests, forest meadows, shrubs, or abandoned tall grasslands (Jordán, Báldi, Orci, Rácz, & Varga, 2003; Krištín & Kaňuch, 2013; Löffler & Fartmann, 2017; Nagy, Rácz, & Varga, 1999; Rácz, 1998).

We could hypothesize that there is weak premating isolation between these two related *Pholidoptera* species, and their rare coexistence results from the selection of different niches, which prevents hybridization and shapes a mosaic pattern of sympatry (Cole, 2016; Gröning et al., 2007). However, if the two species prefer a similar landscape type and climate niche but do not co‐occur as evidence suggests, we alternatively hypothesize that reproductive interference instead, characterized by poaching on nuptial gifts and incorrect sperm investment, may have led to the demographic displacement of species (Kyogoku, 2015). In the first instance, we mapped the occurrence of both species in Slovakia to obtain detailed information on their distribution and environment. Subsequently, using behavioral trials, we tested the possibility of heterospecific mating and whether this behavior also involved the successful transfer of sperms and consumption of the alien nuptial gift. In the present study, we thus aimed to obtain a better understanding of how *P. aptera* and *P. transsylvanica* current distribution patterns provide evidence for a role of reproductive interference and to learn whether there is a prezygotic isolating barrier between these two closely related bush‐crickets.

## 2 MATERIAL AND METHODS

### 2.1 Species mapping

**Figure 2:**
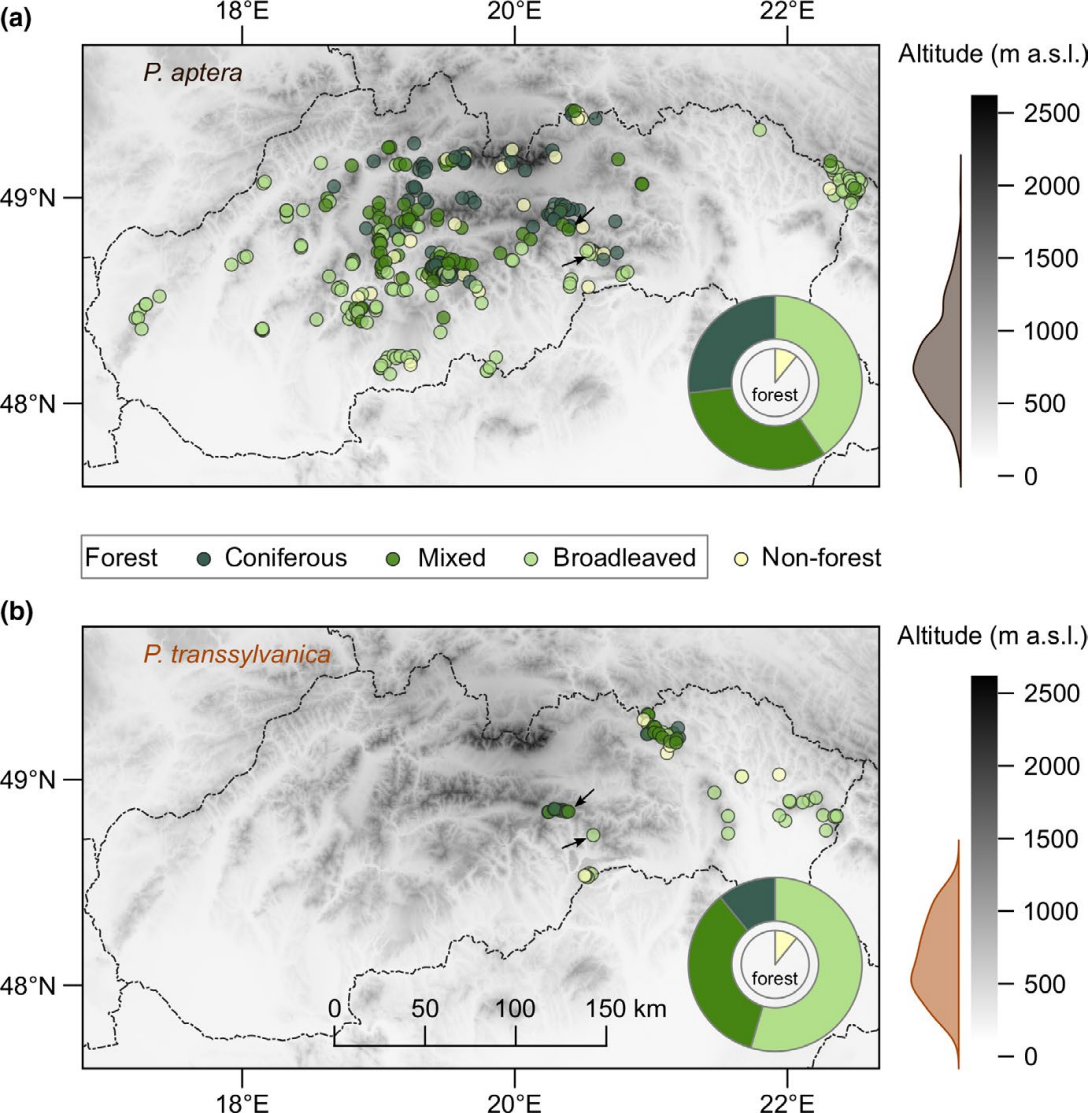
Distributions of *Pholidoptera aptera* (a) and *Pholidoptera transsylvanica* (b) in Slovakia. The dominant habitat of the surrounding landscape around sites within a 1,000 m radius is color‐labelled according to CORINE land cover inventory data with overall proportions in pie charts. Altitudes of sites are summarized in density plots. Arrows point to the two sites of current species coexistence

Distribution maps of *P. aptera* and *P. transsylvanica* in Slovakia (Western Carpathians) were based primarily on our own field work during 1994–2018 (~90% of records) and supplemented by collated data from relevant published sources since the 19th century (Appendix S1). Field collections were conducted from June to September (in average 50 days of field work per year with the help of contributions from more than 80 coworkers) and aimed to sample most of the area during different seasons. For our spatial sampling, we visited 99% of the 10 × 10 km squares of the Universal Transverse Mercator coordinate system that cover the target area and mapped ~1,700 potential sites (http://www.orthoptera.sk). The presence of the study species was recorded mostly by acoustic registration of species‐specific calls of stridulating males, with subsequent catching of individuals using entomological hand nets and their morphological determination (Harz, 1969). We geo‐referenced 331 and 60 occurrence sites of *P. aptera* and *P. transsylvanica*, respectively (Figure 2).

### 2.2 Environmental analyses

The CORINE Land Cover 2012 inventory (European Environment Agency, https://land.copernicus.eu) providing consistent information on land cover was used to characterize the dominant habitat type of the surrounding landscape within a 1,000 m radius around geo‐referenced occurrence sites of *P. aptera* and *P. transsylvanica* (Figure 2). The selected radius was used due to the potential seasonal dispersal range for these large species, according to observed daily movement distances of similar or even smaller ground‐dispersing bush‐crickets (Benedek, Nagy, Rácz, Jordán, & Varga, 2011; Berggren, 2004; Diekötter et al., 2007; Kindvall, 1999; Lorch, Sword, Gwynne, & Anderson, 2005). A principal component analysis (PCA) of climate variables extracted for each site was used to define the climate niches of the species. To reduce colinearity between the 16 climate variables from WorldClim database (https://www.worldclim.org; Hijmans, Cameron, Parra, Jones, & Jarvis, 2005), we retained seven variables with a Pearson's pairwise correlation coefficient of less than |0.8|: annual mean temperature, mean diurnal range, isothermality, precipitation of driest quarter, precipitation of coldest quarter, precipitation of driest month, and precipitation seasonality.

Geo‐spatial analyses for landscape characterization were processed in the QGIS 3.4 software (https://qgis.org). Difference tests of the habitats proportions (Pearson's chi‐square test), sites altitudes (Mann–Whitney *U* test), and PCA of climate niches were performed using the default packages of the R 3.4.4 environment for statistical computing (R Core Team, 2018).

### 2.3 Behavioral experiment

Behavioral sequence of mating in the study species begins with attraction of a female by male's stridulation. Then, the male may be accepted or refused depending on the female's preferences (Gwynne, 2001). Prior to copulation, either a male approaches under the female's abdomen or the female climbs on the male's back, while during the copulation the male grasps the female's ovipositor with its cerci or legs. After copulation, the male attaches to the female's genital opening a spermatophylax (Figure 1). The acoustic characteristics of *P. aptera* and *P. transsylvanica* are similar and vary mainly in echeme repetition rate (Orci, 2002). To obtain evidence of how sexual activity of the two species overlaps in time, we recorded stridulation of multiple captive males during four 45 min sessions at the same time in calm and unclouded summer weather. Acoustic recordings were made using an Edirol R‐09HR (Roland, Inc.) digital recorder (sampling frequency of 96 kHz and 16‐bit amplitude resolution). Oscillographic and spectrographic analyses in the software Audacity 2.2.1 confirmed that during the highest measured activity of males at 16:00 and 20:00, the species almost fully overlapped, because both stridulated persistently (Figure S2).

The hypothesis that reproductive interference may have led to the demographic displacement of the inferior species was tested in a mating experiment with mixed groups of conspecifics and heterospecifics. In early June 2017, bush‐crickets in nymphal stages were collected at two sites in southeastern Slovakia where the absence of coexistence was confirmed (*P. aptera*, 48.585°N, 20.408°E; *P. transsylvanica*, 48.540°N, 20.536°E). Virginal individuals were housed in glass containers 40 × 20 × 20 cm in size with wire netting on top (maximum 4 individuals per one container) and reared for 2 weeks until they fully developed and sexual maturity was ensured. The containers were equipped with a broad‐spectrum 25 W daylight lamp concentrated at a wavelength of 700–900 nm UV‐A with a neodymium sleeve Day Glo (Exo‐Terra, Inc.) to contribute to the insects' physiological well‐being. Prior to our experiment, using a set of adult individuals (18 *P. aptera* and 17 *P. transsylvanica*) in room conditions, we successfully verified that these two species are willing to heterospecific mating in the absence of conspecific mate (10 heterospecific copulations were observed). The behavioral experiment, with mixed mating groups and which lasted 14 days (16–30 June 2017), was then run under open‐air conditions (300 m a.s.l.; average daily temperature 20.3°C, range 13.7–23.1°C; average relative humidity 67%, range 58%–96%) with individuals housed in collapsible insect cages, 55 cm in each dimension, made from white polyester netting, which provided natural temperature and ventilation for the bush‐crickets. Each mating group comprised two unmated adult males and females of each species. In total, 56 individuals were divided into seven mating groups. Rearing cages were checked continuously every 4 hr (i.e., six times per day) to record every possible copulation event. This period was found by previous observations to be the routine time that they needed to finish eating the spermatophylax meal after copulation, and it is similar to other such tettigonids (Gwynne, 2001). Females were individually labelled on the top of their shield with a nontoxic permanent marker, and the date and time of copulations were recorded. When we found a female with a spermatophore or its remnants, up to ~50 mm^3^ of spermatophylax volume (<10% of the total volume) was cut off with sterilized surgical scissors and stored in 96% ethanol for DNA extraction of the respective male donor of this nuptial gift. We aimed to leave most of the spermatophylax for females to freely consume to minimize the effect on their next choice. Difference in the proportion of conspecific to heterospecific copulations between species was tested using the Pearson's chi‐square test. Differences in the length of the refractory period after conspecific and heterospecific copulations were tested using the Mann–Whitney *U* test.

During the whole experiment, bush‐crickets were fed with ground dry cat food, pollen, oat flakes, and fresh leaves of European dewberries. The interiors of the containers and cages were sprayed with water every day (usually in the morning) to provide moisture and water for drinking. Besides bunches of dewberries, individuals could also hide under egg cartons to avoid direct light and reduce stress in captivity. At the end of the experiment, females were euthanized and under a binocular enhancer we dissected spermatodoses from their spermathecas. These spermatophore‐like structures, which are formed to envelope the male ejaculate after copulation, remain within the spermatheca for the duration of the female's life. Using this method, we could verify whether heterospecific sperms were successfully transferred into the female reproductive system, as each spermatodose represents one copulation (Gwynne, 2001; Vahed, 2003).

### 2.4 PCR‐based identification of spermatophores

**Figure 3:**
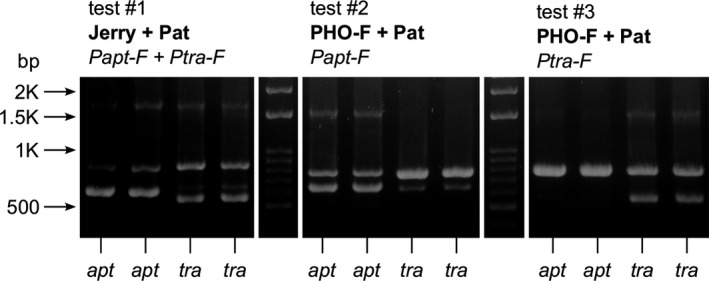
Amplification of COI mtDNA fragments of *Pholidoptera aptera* (*apt*) and *Pholidoptera transsylvanica* (*tra*) spermatophores on 1.5% agarose gel. DNA was amplified using the external (bold) and the species‐specific internal (italic) primers combined in three different determination tests

Identification of the spermatophores of the two *Pholidoptera* species was performed using multiplex PCR and agarose gel electrophoresis. DNA extraction from spermatophylax mass was conducted according to the salting‐out protocol (Aljanabi & Martinez, 1997) modified by added RNaseA (Hornett & Wheat, 2012). In addition to forward Jerry (C1‐J‐2183) and reverse Pat (TL2‐N‐3014) primers (Simon et al., 1994), which amplify a fragment of the COI mtDNA gene, another external forward primer PHO‐F was designed on the basis of homology of known *Pholidoptera* sequences deposited in GenBank (access. no. KY554963–64). Fragments of 750–850 bp amplified with external primers PHO‐F/Pat or Jerry/Pat, respectively, were screened for sequences of 20–23 nucleotides characterized by a maximum of one substitution within species but differing by at least four mutations from the other species (GC content 40%–50%). At these regions, we designed internal forward primers Papt‐F for *P. aptera* and Ptra‐F for *P. transsylvanica* (Table 1), which should produce shorter species‐specific PCR products of 550–650 bp (Figure 3). We prepared three combinations of primers. The first combination (test #1) used 0.4 μM of external forward primer (Jerry), 0.6 μM of external reverse primer (Pat), and 0.2 μM of each internal forward primers Papt‐F and Ptra‐F and 1 × PCRBIO HS Taq Mix master mix (PCR Biosystems) in a 12‐μl reaction volume, including 3 μl of DNA template (5–10 ng/μl), while other combinations used 0.2 μM of external forward primer (PHO‐F), 0.4 μM of Pat and 0.2 μM of Papt‐F (test #2) or 0.2 μM of PHO‐F, 0.4 μM of Pat, and 0.2 μM of Ptra‐F (test #3). COI fragments were amplified in a PCR thermocycler (Biometra TAdvanced) in the following steps: initial denaturation at 95°C for 5 min, 35 cycles of denaturation at 95°C for 30 s, annealing at 47°C for 30 s and extension at 72°C for 90 s, and a final elongation step at 72°C for 5 min. Bands of PCR products were separated on 1.5% agarose gel and visualized by HydraGreen DNA dye. The specificity and efficacy of this rapid and easy discriminating molecular method were verified using the set of adult bush‐crickets. Correct determination of each spermatophore species identity was always secured by test #1 and test #3, respectively, which yielded distinct pattern of bands of the gel (Figure 3).

**Table 1:** Primers used in this study (e—external, i—internal/f—forward, r—reverse)

Primer name	Position	Sequence 5′–3′
Jerry (Simon et al., 1994)	e/f	CAACAYTTATTTTGATTYTTTGG
Pat (Simon et al., 1994)	e/r	TCYAATGCAYTAATCTGCCATATTA
PHO‐F	e/f	GAAAGAGGAAAAAAGGAAGC
Papt‐F	i/f	CCTCAGCCACTATGATTATTGC
Ptra‐F	i/f	CACTTATAGTCCCGCCTTAC

## 3 RESULTS

### 3.1 Distribution, landscape type, and climate niche

**Figure 4:**
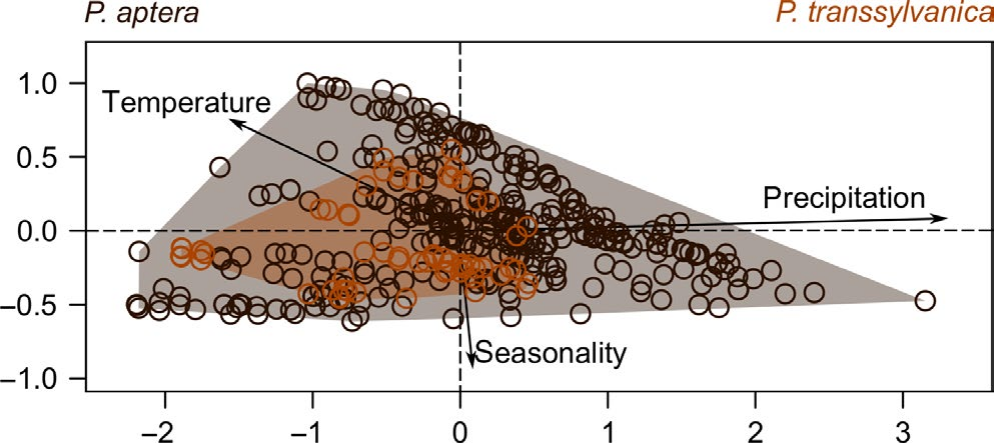
Biplot of *Pholidoptera aptera* and *Pholidoptera transsylvanica* climatic niches constructed from seven climatic variables using PCA. For the sake of clarity, only three variables are presented (temperature—annual mean temperature, precipitation—precipitation of the driest and the coldest quarter, and seasonality—precipitation seasonality)

Surveying the occurrence of bush‐crickets in Slovakia, we found a mostly nonoverlapping mosaic distribution pattern in the two *Pholidoptera* species. The range of *P. aptera* covered most of mountains in the Western Carpathians, whereas the occurrence of *P. transsylvanica* was delimited to the mountain regions of eastern Slovakia, apart from the easternmost area, which was inhabited by the other species. The recent coexistence of the species was found at two sites, which only represents 0.5% of all 391 sites (Figure 2). Regarding landscape type, mostly forests surrounded both *P. aptera* and *P. transsylvanica* sites, 88% and 87%, respectively (Figure 2), while the proportions of dominant forest habitats did not differ significantly between these species (*χ* ^2^ = 7.79, *df* = 6, *p* = .25). The moderately higher representation of coniferous (mostly Norway spruce) forests around the *P. aptera* sites is likely related to the regional topography of the region where it occurs. Comparing to regions of *P. transsylvanica*, occurrence sites of *P. aptera* were located in higher altitudes (*Z* = 3.42, *p* < .001). The first PCA axis explained 83.5% and the second 13.5% of the climatic data variance. The climate niche of *P. transsylvanica* was in complete overlap with *P. aptera* (Figure 4), and its relative breadth was most likely related to the size of distributional area sampled (Figure 2).

### 3.2 Heterospecific mating

**Figure 5:**
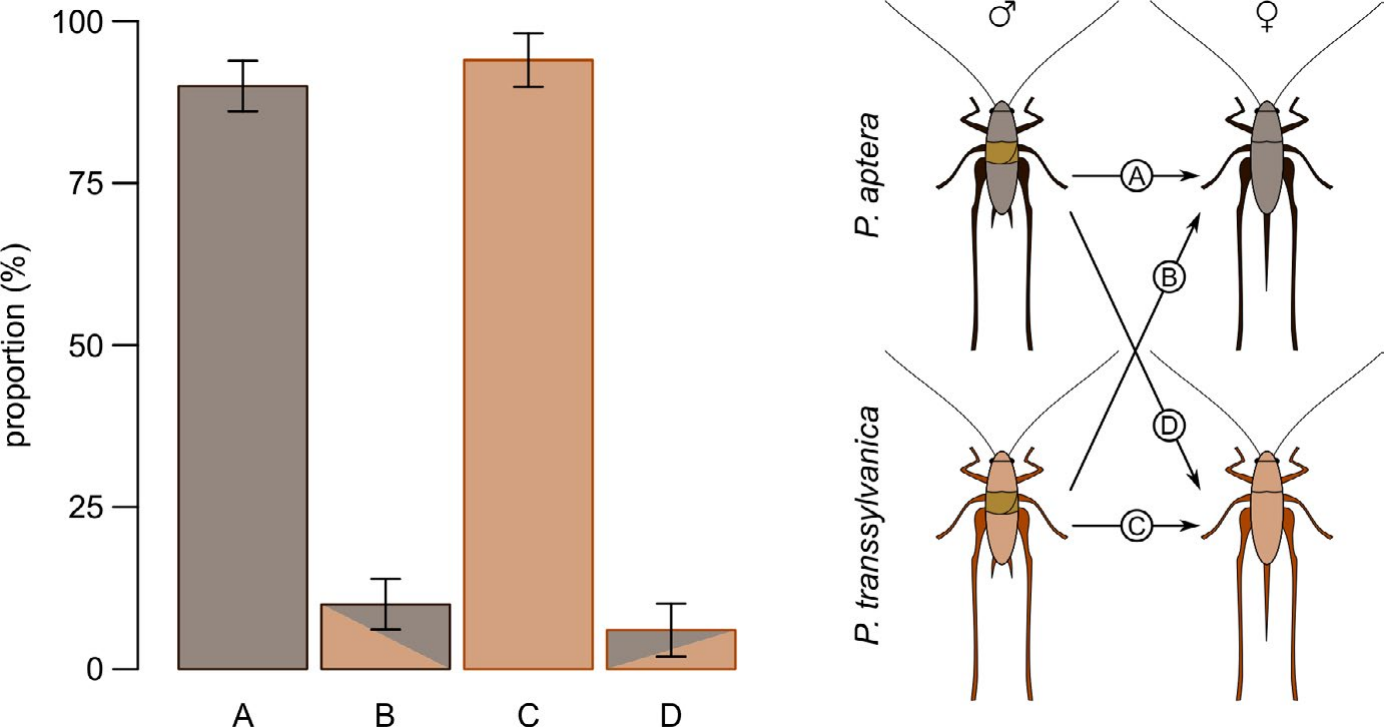
Results of the behavioral experiment. Relative proportion (mean ± *SE*) of mating frequency between conspecifics and heterospecifics in mixed mating groups (*n* = 7 groups of two males and two females of each *Pholidoptera aptera* and *Pholidoptera transsylvanica*) as determined by DNA of transferred spermatophores (*n* = 99)

The mating experiment with mixed groups of conspecifics and heterospecifics provided evidence about possible heterospecific mating choice in the two *Pholidoptera* species. During 14 days in open‐air cages, the average number of received spermatophores was 4.1 (range 3–6) in *P. aptera* and 2.9 (range 1–7) in *P. transsylvanica* females, respectively. In total, 8.1% of copulations were heterospecific (total number of copulations = 99), while females of *P. aptera* admitted 10.3% (*n* = 58) and *P. transsylvanica* 4.9% (*n* = 41) of alien spermatophores. Although copulation of a *P. transsylvanica* male with a *P. aptera* female in the mixed mating groups was slightly more frequent than in the reverse case (Figure 5), proportions of different type of copulations did not differ between species (*χ* ^2^ = 0.37, *df* = 1, *p* = .54). Each female which took part in heterospecific copulation also copulated with conspecifics. Two heterospecific copulations were found in only a single *P. aptera* female, which had five copulations in total. Heterospecific copulations occurred randomly regarding the order of mating events of individual females. We also did not find a significant difference (*Z* = 0.01, *p* = .99) in the length of refractory period that occurred after conspecific (median 3, range 0–6 days) and heterospecific copulation (median 3, range 2–3 days).

Along with conspecific, we found also heterospecific spermatodoses in the females' spermathecas, which confirmed that heterospecific sperms were successfully transferred into the female's reproductive system during copulation. The number of spermatodoses was identical to the number of recorded spermatophores in all females.

## 4 DISCUSSION

By merging field‐based evidence with experimental testing, we suggested reproductive interference in two related bush‐crickets, *P. aptera* and *P. transsylvanica*, where analyzed climatic and environmental factors likely did not play a significant role in the shaping of their geographical distributions in Central Europe. Instead, we found that the two species do not differ in either the landscape type or climate niche they occupy, but that they can engage in heterospecific mating if they come into contact.

The observed distribution pattern suggests that the species have colonized the study area at the northern margin of their ranges from different refugia, which favors the imperfect premating isolation between them (Gröning & Hochkirch, 2008; Tregenza, 2002). The range of *P. aptera* is typical for postglacial recolonization of Central Europe from the Balkans, whereas the restricted distribution of *P. transsylvanica* in Carpathian Mountains has obviously a relict character (Kenyeres, Rácz, & Varga, 2009). We also found that the two species only coexist in two sites in Western Carpathians in Slovakia, supporting species‐specific colonization histories but also a contemporary contact zone with a mosaic distribution pattern (Figure 2). Although there is no evidence about the utilization of different resources (e.g., prey) by these two species, premating isolation in this zone could be facilitated by spatial or temporal barriers to gene flow, which arise as by‐products of ecological divergence (Eroukhmanoff, Hargeby, & Svensson, 2011; Kádár et al., 2015). There could also be some habitat differentiation at the local scale, as indicated by the larger distance of *P. transsylvanica* from the forest edge toward a warmer microclimate than in *P. aptera* (Nagy et al., 1999 and own unpublished data), leading to a possible shift in the phenological development of the eggs (missing data) between species (earlier hatching of nymphs is expected in *P. transsylvanica*). However, none of these mechanisms can physically separate individuals and thus prevent reproductive interference at common sites (Jang & Gerhardt, 2006).

The females of *P. transsylvanica* in our experiment admitted twice as less alien spermatophores than *P. aptera*, and we can speculate how much of this proportion is responsible for the demographic displacement of one of the species. If the proportion of heterospecific mating is similar under field conditions, from the long‐term perspective we believe that permanent poaching on heterospecific spermatophores—which is in a way a form of resource competition—may substantially limit the reproductive success of the less frequent *P. transsylvanica* in the area (Hochkirch et al., 2007). In fact, because one of the older records by Chládek (2003) is no longer a coexistence site and *P. transsylvanica* is the species that was displaced, this could be evidence of exclusion by reproductive interference. However, the effect of temporary habitat change should be considered in that case also.

Although males can control the protein composition of the spermatophylax, both qualitatively and quantitatively, according to traits of the receiving female (Jarrige, Body, Giron, Greenfield, & Goubault, 2015), preliminary evidence on the protein diversity of the spermatophylax suggests that males provide basic nutrients in nuptial gifts to deter females from removing the spermatophore until successful sperm transfer has happened, rather than to chemically manipulate female postcopulation behavior (Lehmann et al., 2017). At least, we have no evidence that heterospecific mating would somehow affect the length of the refractory period. Bush‐cricket females have larger parental investment than males, and their mate choice is therefore also influenced by direct nutritional benefits (Gwynne, 1990; Lehmann, 2012). Occasional mating with heterospecific males should depend on their size, because if they are larger than conspecifics they should provide larger nuptial gifts (Costa‐Schmidt & Machado, 2012; Dorková, Naďo, Jarčuška, & Kaňuch, 2019). We, therefore, suggest that in the case of interspecific mating interactions between *P. aptera* and *P. transsylvanica* the female's choice of large males is what drives reproductive interference. Consequently, *P. aptera* females may benefit more from interspecific mating than *P. transsylvanica* females, which may increase their relative fitness. Moreover, larger *P. transsylvanica* males can outperform smaller *P. aptera* competitors, and also *P. transsylvanica* females can easily reject a forced heterospecific copulation attempt from *P. aptera* males (Ribeiro & Spielman, 1986). However, a measure of the net costs of heterospecific mating for females is clearly needed. A little is also known about inter‐ and intra‐specific variation in the shape of males' titillators (Harz, 1969). Since these sclerotized genital appendices are used in female stimulation and spermatophore transfer, it would be also interesting to determine the possible role of this sexual trait in heterospecific female mate choice (Vahed, 2015; Wulff et al., 2015). The costs of heterospecific mating are particularly high if postmating barriers are complete (e.g., gametic incompatibility or zygotic mortality), and there is no chance of siring viable hybrids (Gröning & Hochkirch, 2008). As we confirmed the successful transfer of heterospecific sperms into females' spermathecas, further research is needed to investigate whether these two species are fully reproductively isolated, especially at sites where they coexist.

## CONFLICT OF INTEREST

None declared.

## AUTHOR CONTRIBUTIONS

P.K. and A.K. conceived the idea and designed the study. A.K. performed most of the field work; M.D., A.K., and B.J. conducted experiment; M.D. carried out the molecular work. B.J. and P.K. performed analyses. P.K. drafted the manuscript. All authors contributed to writing the final version of the manuscript.

## Data Availability

Geographical coordinates of the distributional data are available from the Dryad Digital Repository: https://doi.org/10.5061/dryad.vmcvdncpk.
