# Supplementary figures and images for: The mosaic distribution pattern of two sister bush‐cricket species and the possible role of reproductive interference
Source: Ecol Evol. 2020 Feb 8;10(5):2570–8. doi: 10.1002/ece3.6086 (PMC7069280; doi:10.1002/ece3.6086)

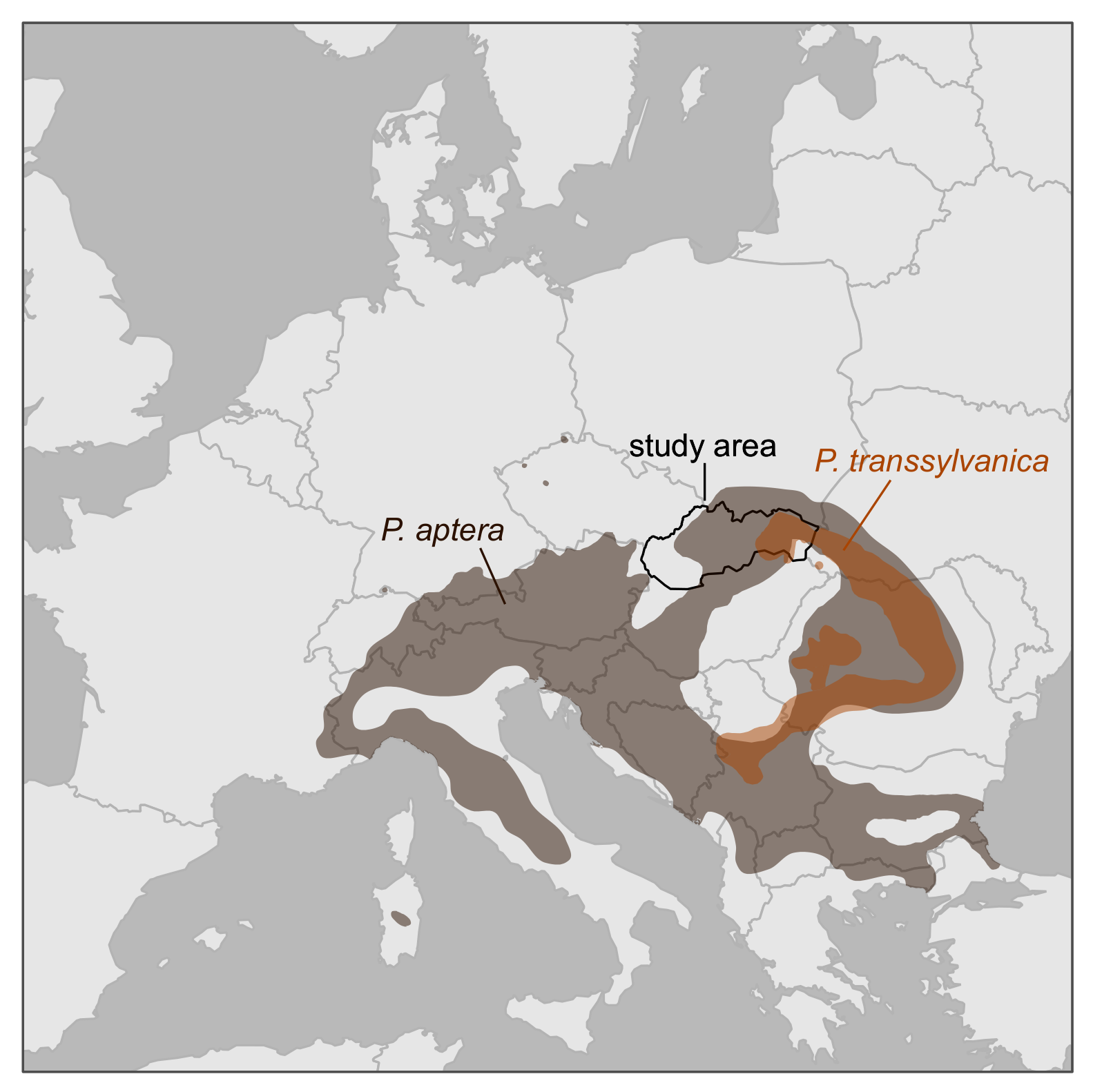

Supplement: Supplementary file 1 [file ECE3-10-2570-s001.tif]

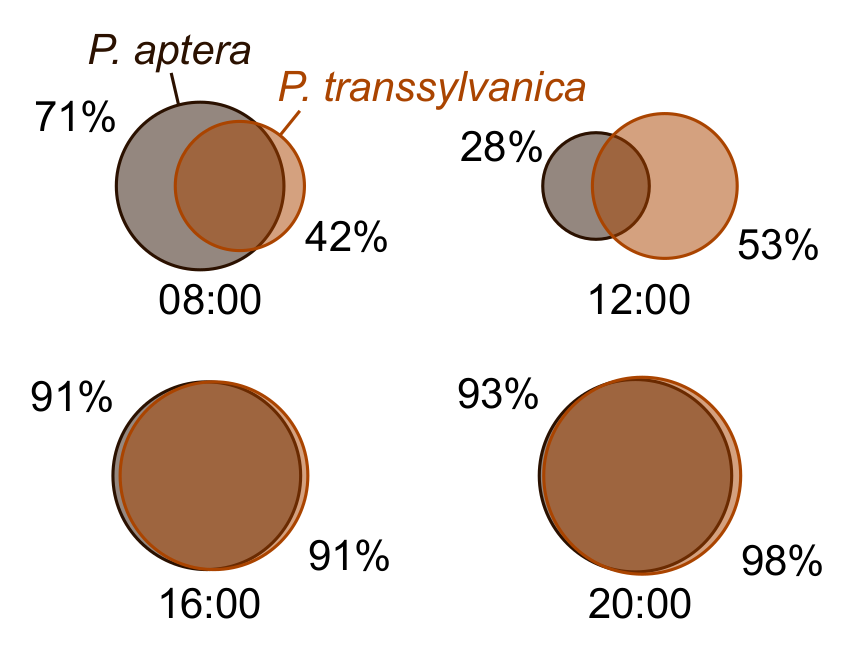

Supplement: Supplementary file 2 [file ECE3-10-2570-s002.tif]
