# Supplementary material for: The mosaic distribution pattern of two sister bush‐cricket species and the possible role of reproductive interference
Source: Ecol Evol. 2020 Feb 8;10(5):2570–8. doi: 10.1002/ece3.6086 (PMC7069280; doi:10.1002/ece3.6086)
Supplement: Supplementary file 3 [file ECE3-10-2570-s003.doc]

Appendix S1. The list of references of species records in Slovakia

Čejchan A (1958) Příspěvek k poznání rovnokřídlého hmyzu (Orthoptera) Slovenska II. Beitrag zur Kenntnis der Orthopteren von Slovakei II. Časopis Slezského muzea, Series A – Historia naturalis, 7, 1–7.

Čejchan A (1987) Poznámky o orthopteroidním hmyzu (s.l.) Slánských vrchů (Grylloptera, Orthoptera s.str., Dictyoptera - Blattodea). Sborník Národního muzea Praha, Přírodní vědy, 43, 59–62.

Čejchan A (1988) K poznání orthopteroidního hmyzu (s.l.) Nízkých Tater (Grylloptera, Orthoptera s.str., Dermaptera, Dictuoptera: Blattodea). Sborník Národního muzea Praha, Přírodní vědy, 44, 1–9.

Čejchan A (1989) K poznání orthopteroidního hmyzu (s.l.) Bukovských vrchů v CHKO Východné Karpaty (Grylloptera, Orthoptera s.str., Dermaptera, Dictuoptera). Sborník Národního muzea Praha, Přírodní vědy, 44, 65–74.

Čejchan A (1993) Orthopteroidní hmyz (s.l.) CHKO Muráňská planina (Slovensko). Orthopteroid insects (s.l.) of the Protected Landscape Area Muráň Plan (Slovakia). Časopis Národního muzea Praha, Řada přírodovědná, 161, 47–56.

Frivaldszkyi J (1867) Monographia Orthopterorum Hungariae. Eggenberger, Pest.

Chládek F (1968) Příspěvek k rozšíření rovnokřídlého hmyzu (Saltatoria) v Československu. Zpravodaj Československé společnosti entomologické ČSAV, 4, 47–49.

Chládek F (1986) K vertikálnímu rozšíření rovnokřídlých (Orthoptera), švábů (Blattoptera) a škvorů (Dermaptera) v Belianských Tatrách. Zpravodaj Československé společnosti entomologické ČSAV, 22, 103–108.

Chládek F (1994) Rovnokrídlovce (Orthoptera), šváby (Blattoptera), modlivky (Mantoptera) a ucholaky (Dermaptera). Pp. 157–163. In: Rozložník M, Karasová M (eds), Slovenský kras. Chránená krajinná oblasť – biosferická rezervácia. Osveta, Martin.

Chládek F (1999) K poznání rovnokřídlých (Orthoptera s.l., Insecta) Slovenska. Tetrix, 1, 25–31.

Chládek F (2003) Zweiter Beitrag zur Kenntnis der Geradflügler (Orthoptera s. l., Insecta) in der Slowakei. Tetrix, 10, 58–60.

Gavlas V (1999) K poznaniu rovnokrídlovcov (Ensifera, Gryllodea, Caelifera) a modliviek (Mantodea) južnej časti Strážovských vrchov. Folia faunistica Slovaca, 4, 55–64.

Gavlas V (2005) Orthoptera species of European importance in Slovakia. Articulata, 20, 57–68.

Gulička J (1967) Orthoptera, Blattodea, Mantodea, Dermaptera zátopového územia pod Vihorlatom. Acta Facultatis rerum naturalium Universitatis Comeniane, Zoologica, 12, 41–62.

Holuša J (1996) A contribution to the knowledge of the distribution of grasshoppers and crickets throughout Slovakia. Entomofauna carpathica, 8, 115–124.

Mařan J (1953) Příspěvěk k poznání rodu *Pholidoptera* Wesm. Acta entomologica musei nationalis Pragae, 28, 209–221.
