# Supplementary material for: The mosaic distribution pattern of two sister bush‐cricket species and the possible role of reproductive interference
Source: Ecol Evol. 2020 Feb 8;10(5):2570–8. doi: 10.1002/ece3.6086 (PMC7069280; doi:10.1002/ece3.6086)
Supplement: Supplementary file 4 [file ECE3-10-2570-s004.docx]

**Supporting Information**

Fig S1. Species ranges in Europe by the “species extent of occurrence” (source: Chobanov et al. 2016, Hochkirch et al. 2016).

Fig. S2. Relative duration of *P. aptera* and *P. transsylvanica* males’ stridulations and their temporal overlap during 45 min sessions on a different time of day (start in CEST).
